# mHealth in Urology: A Review of Experts’ Involvement in App Development

**DOI:** 10.1371/journal.pone.0125547

**Published:** 2015-05-18

**Authors:** Nuno Pereira-Azevedo, Eduardo Carrasquinho, Eduardo Cardoso de Oliveira, Vitor Cavadas, Luís Osório, Avelino Fraga, Miguel Castelo-Branco, Monique J. Roobol

**Affiliations:** 1 Faculty of Health Sciences, University of Beira Interior, Covilhaã, Portugal; 2 Department of Urology, Espírito Santo Hospital, Évora, Portugal; 3 Department of Urology, Porto Hospital Centre, Porto, Portugal; 4 Erasmus University, Erasmus Medical Centre, Rotterdam, The Netherlands; Oklahoma University Health Sciences Center, UNITED STATES

## Abstract

**Introduction:**

Smartphones are increasingly playing a role in healthcare and previous studies assessing medical applications (apps) have raised concerns about lack of expert involvement and low content accuracy. However, there are no such studies in Urology. We reviewed Urology apps with the aim of assessing the level of participation of healthcare professionals (HCP) and scientific Urology associations in their development.

**Material and Methods:**

A systematic search was performed on PubMed, Apple's App Store and Google's Play Store, for Urology apps, available in English. Apps were reviewed by three graders to determine the app’s platform, target customer, developer, app type, app category, price and the participation of a HCP or a scientific Urology association in the development.

**Results:**

The search yielded 372 apps, of which 150 were specific for Urology. A fifth of all apps had no HCP involvement (20.7%) and only a third had been developed with a scientific Urology association (34.7%). The lowest percentage of HCP (13.4%) and urological association (1.9%) involvement was in apps designed for the general population. Furthermore, there was no contribution from an Urology society in "Electronic Medical Record" nor in "Patient Information" apps. A limitation of the study is that only Android and iOS apps were reviewed.

**Conclusions:**

Despite the increasing Mobile Health (mHealth) market, this is the first study that demonstrates the lack of expert participation in the design of Urology apps, particularly in apps designed for the general public. Until clear regulation is enforced, the urological community should help regulate app development. Maintaining a register of certified apps or issuing an official scientific seal of approval could improve overall app quality. We propose that urologists become stakeholders in mHealth, shaping future app design and promoting peer-review app validation.

## Introduction

Smartphones and tablets are almost ubiquitous in our society and represent a popular method of accessing information. Smartphone applications (apps) are increasingly playing a role in healthcare [1]. Mobile Health (mHealth) comprises "medical and public health practice supported by mobile devices" [2]. The total mHealth market revenue is estimated to grow by about 61% to reach US$26 billion, at the end of 2017 [3]. Moreover, previous studies report close to 100,000 medical apps available on the two leading software platforms, iOS (Apple) and Android (Google) [3]. That number is expected to grow even further, as both Apple and Google have announced mHealth to be a top-priority [4, 5]. With the increasing number of available apps, there is a growing concern about their quality and safety, as there are no industry standards, no scientific guidelines and no independent medical app regulation [6–8].

Recently, papers assessing apps in various medical fields have been published, detailing the myriad of options available, which range from health and fitness apps for the general public, to medical education and teaching aids, as well as electronic health records and even augmented reality software [1, 9–12]. The clinical use of smartphone as diagnostic tools in Dermatology is one of the most common, but it is not without pitfalls: an app that claimed to quantify skin cancer risk mislabeled 80% of textbook melanomas [13, 14].

Apps designed for surgical specialties, such as Anesthesiology, Plastic Surgery and Neurosurgery, have also been scrutinized, with similar conclusions: app development offers great potential, but lacks standardized regulatory procedures [15–17]. Even though there have been papers demonstrating the use of apps in Urology, to our knowledge, there are no published studies reviewing healthcare professional involvement in apps specifically designed for Urology [18, 19]. This study had two aims. First, to review Urology apps, mainly in regards to mobile platform, target audience, developer, type of app, app category and price. Second, to identify which apps had documented HCP involvement and which were developed in collaboration with a scientific Urology association.

## Methods

Three graders (an Urology resident and two Urology specialists) conducted a systematic review on PubMed, Apple App Store (iOS) and Google Play Store (Android), for Urology-themed apps, between September of 2014 and January of 2015. On PubMed, the searched terms were "Urology" and "mobile", "application", "app", "apps", "mHealth", "eHealth", "iOS", "iPhone", "iPad", "Android", "tablet" and "smartphone". Results were filtered for articles related with Urology apps that were available for download on the Apple App Store and on Google Play Store.

A similar query was also performed on both stores, for Urology-related apps. Android apps were searched at the Google Play online store. For iOS-based applications, the search was performed using iTunes v11.3.1 for Mac OS X (Apple Inc., Cupertino, CA, USA). All apps with "Urology" in their metadata (title, description, keywords and version history) were scrutinized. From the search results, graders included all apps that were available in English and designed specifically for Urology (e.g. "AUA Core Curriculum Mobile"). Exclusion criteria include those related with general medicine (e.g. "Clinical Tests & Procedures") or other fields (e.g. "Anatomy Flash Cards"), and apps that only had product advertisement, i.e. apps that only promoted pharmaceutical or medical equipment (e.g. "Actient Pharmaceuticals"). All three graders universally agreed on the criteria.

Based on all available information, the three reviewers decided on one of the following apps' type: Reference, Guidelines, and Quiz/Exam (e.g. Urology textbooks, Guidelines from an urological association); Conferences, Urological Societies/Associations, Journals and Institutions (e.g. "EAU Stockholm 2014", "AUA Member Search"); Calculators (e.g. "TNM Urology"); Electronic Medical Record/Diaries (e.g. "Bladder Pal"); and Patient Information (e.g. "Dealing with Prostate Cancer") (Table 1). They also determined the target audience (i.e. apps designed specifically for healthcare professionals or suitable for the general public). Moreover, they gathered data on the type of developer (i.e., apps developed by an individual or an organization) and the app's category. The app's category is chosen by the developer from a predetermined list of options, and represents the category where the app is available in the Store. Developers are required to select the category that best describes the app. Possible categories are Medical, Books & Reference, Education, Health & Fitness, Business, News & Magazines, Social, Utilities, Entertainment and Games. The app price (free or paid) and the actual price in dollars were also recorded.

**Table 1:** List of all included Urology apps.

*App Name*	*Mobile Platform*	*Scientific Urology Society involvement*	*Healthcare Professional involvement*
@Hand: Urology	iOS	No	Yes
28 Congreso de Urologia 2014	Android	Yes	Yes
3 Prostate Diseases (Tanzania)	Android	No	No
Abnormal Urine Guide	iOS	No	No
Advanced Urology	Android and iOS	No	No
AUA 2011 Courses	Android	Yes	Yes
AUA 2012 Annual Meeting	Android	Yes	Yes
AUA 2013 Annual Meeting	Android and iOS	Yes	Yes
AUA 2014 Annual Meeting	Android and iOS	Yes	Yes
AUA Annual Meeting	Android	Yes	Yes
AUA Core Curriculum Mobile	Both	Yes	Yes
AUA EBJC—Evidence-Based JC	Android	Yes	Yes
AUA Guidelines at a Glance	Android	Yes	Yes
AUA Medical Student Curriculum	Android and iOS	Yes	Yes
AUA Member Search	Android and iOS	Yes	Yes
AUA Men's Health Checklist	Android	Yes	Yes
AURO.it Nazionale 2013	iOS	Yes	Yes
Bedwetting Info	Android	No	No
Bedwetting solutions	Android	No	No
Bedwetting Trainer	Android	No	Yes
BJUI Journal	iOS	Yes	Yes
Bladder Cancer Prognosis Calc	iOS	Yes	Yes
Bladder Pal	Android	No	Yes
Braz J Urol	Android and iOS	Yes	Yes
Briganti Nomogram	Android	No	No
BSC Urology Events	Android and iOS	No	No
CalcuLithiasis	iOS	Yes	Yes
CAU2014	Android	Yes	Yes
CROJ	Android	No	Yes
CRPC Nomogram App	Android	No	Yes
CURE-UAB	Android and iOS	No	Yes
Current Opinion in Urology	iOS	No	Yes
Daily-P	Android and iOS	No	Yes
Daily-P Pro	Android and iOS	No	Yes
Dealing with Prostate Cancer	Android	No	No
Dealing with Prostate Cancer Free	Android	No	No
DGU 2012	Android and iOS	Yes	Yes
DGU 2014	iOS	Yes	Yes
DGU 2014—Kongress App	Android	Yes	Yes
drawMD Female Pelvic Surgery	iOS	No	Yes
drawMD Urology—Patient Education by Drawing on Medical	iOS	No	Yes
DutasT	Android and iOS	No	Yes
e-URO Tools	Android and iOS	Yes	Yes
EAU 2012	Android and iOS	Yes	Yes
EAU Milan 2013	Android and iOS	Yes	Yes
EAU Pocket Guidelines	Android and iOS	Yes	Yes
EAU Stockholm 2014	Android and iOS	Yes	Yes
EAU Vienna 2011	Android and iOS	Yes	Yes
EAUN Milan 2013	Android and iOS	Yes	Yes
EAUN Stockholm 2014	Android and iOS	Yes	Yes
ESPU 2012	Android and iOS	Yes	Yes
ESPU 2013	Android and iOS	Yes	Yes
European Urology app	Android and iOS	Yes	Yes
EurUro SiM	iOS	Yes	Yes
Female Pelvic Medicine & Reconstructive Surgery	iOS	Yes	Yes
Foundation Urology	Android	No	Yes
GU Path I	iOS	No	Yes
GU Path Lite	iOS	No	Yes
HapPee Time	Android	No	No
iCU Evora 2011	iOS	Yes	Yes
iDry	iOS	No	Yes
Int'l Urogynecology Journal	Android	Yes	Yes
iP Voiding Diary	iOS	No	Yes
iReflux Risk Calculator	Android and iOS	No	No
itsaMANTHING—Prostate Cancer	Android	No	No
iURO Andrology	Android and iOS	No	Yes
iURO Andrology PRO	Android and iOS	No	Yes
iURO General Practicioner	iOS	No	Yes
iURO Kidney	Android and iOS	No	Yes
iURO Oncology	Android and iOS	No	Yes
iURO Oncology Pro	Android and iOS	No	Yes
iURO Pelvic Floor	Android and iOS	No	Yes
iURO Pelvic Floor Pro	Android and iOS	No	Yes
iURO Prostate Pro	Android and iOS	No	Yes
Kidney and Bladder Problems	Android and iOS	No	No
Kidney Cancer	Android	No	No
Kidney Disease Assistant	iOS	No	No
Kidney Diseases	iOS	No	No
Kidney Urology—Simulations and behaviours of diseases	iOS	No	Yes
kidneystoneMD	iOS	No	Yes
Learning Urology Quiz	Android and iOS	No	Yes
Male impotence risk evaluation	Android	No	No
male_Japanese	iOS	No	Yes
Men's App	iOS	No	Yes
Men's Guide To Prostate Health	Android	No	No
Miniatlas Erectile Dysfunction	iOS	No	Yes
My BladderDiary	Android	No	No
NMIBC Toolbox	Android	No	Yes
Oxford Handbook Urology 2nd Ed	Android and iOS	No	Yes
Partin/Han Tables	iOS	No	Yes
PI-RADS Prostate MRI	Android	No	Yes
Prac. Urology for Primary Care	Android and iOS	No	Yes
Practical Urology	Android and iOS	No	Yes
Practical Urology for Gynecologists	iOS	No	Yes
Prevent Prostate Cancer	Android	No	No
Primary Care Guidelines for Urology	Android and iOS	Yes	Yes
Prostate Cancer	Android	No	Yes
Prostate Cancer Calculator	Android	No	No
Prostate Cancer Calculator (Seoul National University)	Android	No	Yes
Prostate Cancer v2	Android	No	Yes
Prostate Health	Android and iOS	No	Yes
Prostate In Focus	Android	No	Yes
PROSTATE INTERNATIONAL	Android	Yes	Yes
Prostate Pal 2	Android	No	Yes
ProstateMD	Android	No	Yes
Renal & Urology News	Android and iOS	No	Yes
Reviews in Urology	iOS	No	Yes
Rotterdam Prostate Cancer Risk	Android and iOS	Yes	Yes
POY (Russian Society of Urology)	Android	Yes	Yes
Show Me OAB	iOS	No	No
SIU 2013	Android and iOS	Yes	Yes
SMU 2014	Android	Yes	Yes
Testicle pain, testicle tumors	Android	No	No
Testicular Cancer	Android	No	No
Testicular Cancer Checker	iOS	No	No
The Journal of Urology, Official Journal of AUA	iOS	Yes	Yes
Three Diseases of the Prostate	Android	No	No
TNM Urology	iOS	No	Yes
Turkish Journal of Urology	iOS	Yes	Yes
Understanding Prostate Cancer	Android	No	Yes
UrinaryAlmanac	iOS	No	Yes
Uro Challenge	Android and iOS	Yes	Yes
UroBladderDiary	iOS	No	No
Urolithiasis Assist	Android	Yes	Yes
Urologic Nurse CURN, 800 MCQs	Android	No	Yes
Urologic Oncology: Seminars and Original Investigation	iOS	Yes	Yes
Urological Surgery	Android	No	Yes
Urological Ultrasound	Android	No	Yes
Urology	iOS	No	No
Urology—Pediatric, 1000 MCQs	Android	No	Yes
Urology Board Review Manual	Android and iOS	No	Yes
Urology Case Reports	iOS	Yes	Yes
Urology Flashcards	Android and iOS	No	Yes
Urology for Gynecologists	Android	No	Yes
Urology Glossary	Android and iOS	No	No
Urology Nation	Android and iOS	No	Yes
Urology NBI Atlas by Olympus	iOS	No	Yes
Urology Patient Education by CoherentRx	iOS	No	Yes
Urology Planet	iOS	No	No
Urology Times	Android and iOS	No	Yes
Urology, 1000 MCQs	Android	No	Yes
Urology, The Gold Journal	iOS	Yes	Yes
UrologyMatch	Android and iOS	No	Yes
UroSketch 3D Explore	iOS	No	Yes
UroSketch 3D Professional	iOS	No	Yes
USICON	Android	Yes	Yes
USICON 2014	Android and iOS	Yes	Yes
UWPEN	iOS	No	No
Vasectomy Reversal	Android	No	Yes
WCE 2013 Annual Meeting	Android	Yes	Yes

Complete list of all included apps, its availability and the participation of a HCP or a scientific Urology association. A complete assessment of all urology apps, including its description and information about its creators, is available as supporting information.

Graders considered that there was involvement by HCP (e.g. urologists, other medical doctors, pharmacists, and specialist nurses) or a scientific Urology association in the apps’ design when it was mentioned in the app's description or website. Apps were not purchased or downloaded.

As an example, the app "AUA 2014 Annual Meeting" would be recorded in the database as: platform (Android and iOS), Target (Health professionals), Developer (Organization), Type (Conferences, Urological Societies/Associations, Journals and Institutions), Category (Medical), Price (Free), Actual price (0.0$), HCP involvement (Yes), scientific Urology association involvement (Yes).

First, a descriptive overview of available apps was performed. Second, all apps included in this review were assessed regardless of being available on both platforms or exclusively on Apple App Store or Google Play Store. When an app was available in both Stores, it was evaluated only once. However, some apps available in both platforms had differences between the two versions, namely in price and app category. When the price of the app was not the same on both Stores, the average price was calculated. When the app category was not the same on both platforms, the most recent version was considered.

To evaluate an association between targeted audience and healthcare professional or urological association involvement in the app development, the app price, and the developer, we used the chi-square test of association.

To analyze a relationship between the type of application and the involvement of a healthcare professional or an urological association in the development of the app, we calculated the chi-square test of association.

Analyses were performed using SPSS v20 (IBM Corp., Armonk, NY, USA).

Statistical significance was set at p < 0.05 for all analyses.

## Results

**Fig 1:**
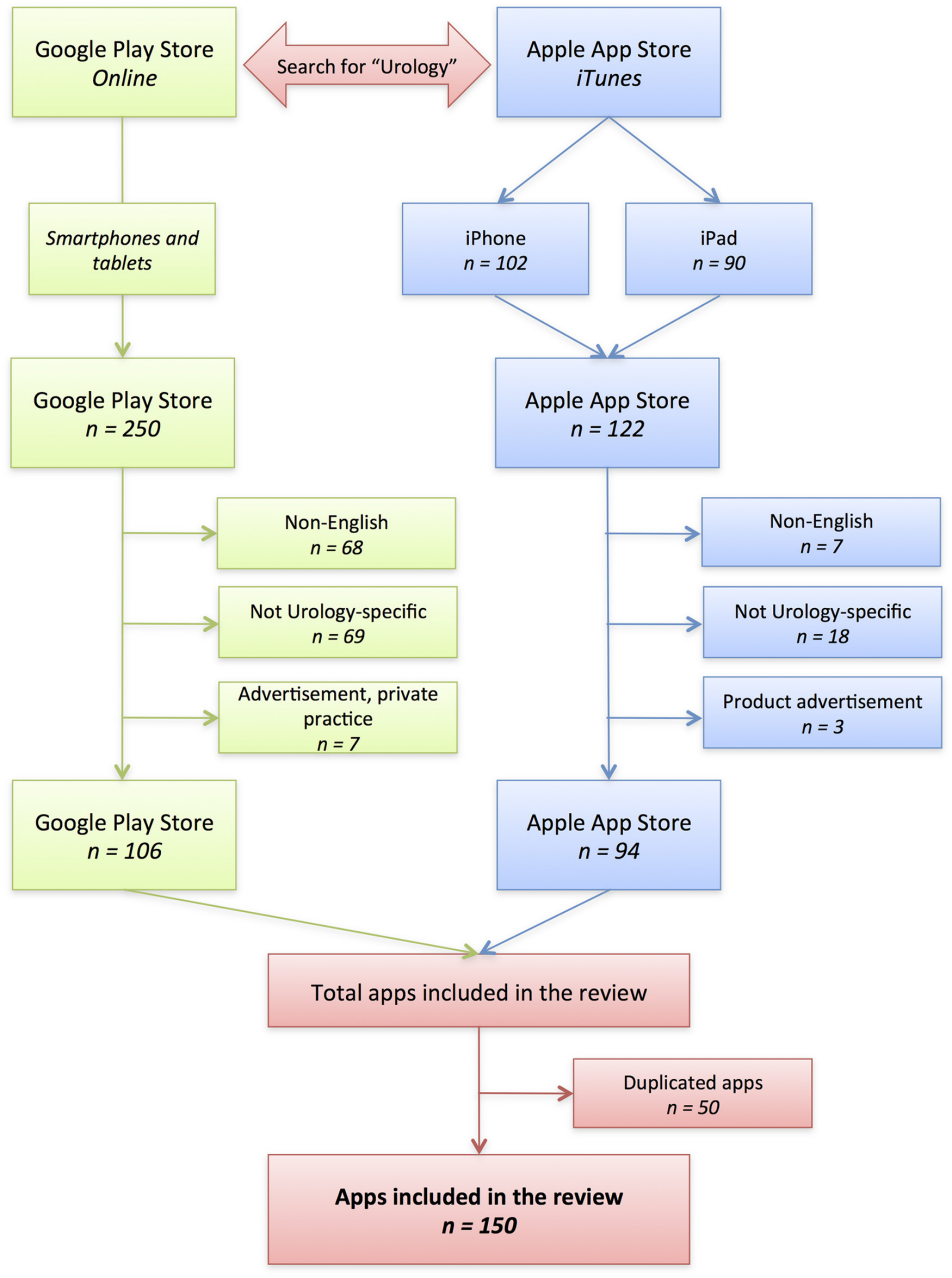
Search methodology for Urology apps. From the initial 372 apps (Android = 250, iOS = 122), we excluded apps not available in English, not specific for Urology or that were only product advertisement, for a total of 150 Urology apps (n = 44 exclusively for iOS, n = 56 exclusively for Android and n = 50 available for both platforms).

From the initial 372 apps (Android = 250, iOS = 122), we excluded all apps that were not available in English, not specific for Urology and that were only product advertisement (Fig 1). Table 1 lists all included apps and Table 2 displays the main characteristics of the surveyed apps. A complete assessment of all urology apps, including its description and information about its creators, is available as S1 Table, and can be accessed at http://dx.doi.org/10.6084/m9.figshare.1363120.

**Table 2:** Summary of descriptive statistics of Urology apps.

Factors	Description	Statistics
Platform^a^	Google	37.3%
	Apple	29.3%
	Both	33.3%
Target audience^a^	Specific for health professionals	72.7%
	Designed for the general public	27.3%
Developer^a^	Individual	12.0%
	Organization (Company/Association/University/Etc.)	88.0%
Apps’ type^a^	Reference, Guidelines, Quiz/Exam	36.7%
	Conferences, Urological Societies/Associations, Journals and Institutions	29.3%
	Calculator	8.7%
	Electronic Medical Record/Diaries	6.7%
	Patient information	18.7%
Apps’ category in the Store^a^	Medical	68.7%
	Books & Reference	9.3%
	Education	8.7%
	Health & Fitness	8.7%
	Business	1.3%
	News & Magazines	0.7%
	Social	0.7%
	Productivity	1.3%
	Games	0.7%
Free or Paid^a^	Free	71.3%
	Paid	28.7%
Actual app price($)^b^	Max price	71.94$
	Minimum price (of paid)	0.99$
	Mean price ± SD for paid apps	$9.15 ± $14.09
	Mean price ± SD for all apps	$2.62 ± $8.55
Involvement of health	No	20.7%
professional in app’ design^a^ ^c^	Yes	79.3%
Involvement of Urological	No	65.3%
Association in app’ design^a^ ^d^	Yes	34.7%

Summary of descriptive statistics of Urology apps and information regarding their platform, target audience, developer type, app type, app category, cost and involvement of a healthcare professional or an urological society.

^a^The percentages frequency distributions are reported for nominal and ordinal variables

^b^The maximum, minimum, and mean values are presented for the actual price.

^c^The involvement of a healthcare professional was assumed if there was reference to an urologist, other medical doctors, pharmacists or specialist nurses in the app.

^d^The involvement of an Urology association was assumed if there was reference to an Urology association.

We found 44 apps exclusive to Apple App Store, 56 uniquely on Google Play Store and 50 available on both stores, for a total of 150 Urology apps.

Of the 150 individual apps, there were more apps targeted at healthcare professionals (72.7%) and published by organizations (88.0%). The most common type of app was "References, Guidelines, and Quiz/Exam" (36.7%), which included, for example, Urology textbooks and atlas.

Regarding app category, most were classified as "Medical" (68.7%), but many were available in the "Books & Reference" section (9.3%).

The vast majority of apps were free (71.3%). The average price of paid applications was 9.15 ±14.09 dollars, but there was a large range, from 0.99 dollars (five apps) to 71.94 dollars ("Urological Surgery", 71.94 dollars). The most expensive apps were "References, Guidelines, and Quiz/Exam". Taking into account the available free apps, the average app price dropped to 2.62 ± 8.55 dollars.

One in five apps had no documented HCP involvement in their design (20.7%). Moreover, only one-third of all reviewed apps had been developed in collaboration with a scientific Urology association (34.7%).

Furthermore, there was a statistically significant difference between target audience and HCP involvement in apps’ design (p<0.001): only 13.4% of all apps designed with input from a HCP were targeted for the general population. Additionally, there was a statistically significant difference between target audience and urological association involvement in the apps’ design (p<0.001). Similarly, the lowest percentage of urological association involvement in apps’ design was in apps designed for the general population (1.9%) (Table 3).

**Table 3:** Association between target audience and expert involvement.

Target audience	Healthcare professional involvement in app’ development			Urological association involvement in app’ development		
	No (0)	Yes (1)	p-value	No (0)	Yes (1)	p-value
Health professionals	6 (19.4%)	103 (86.6%)	0.000	58 (59.2%)	51 (98.1%)	0.000
General	25 (80.6%)	16 (13.4%)		40 (40.8%)	1 (1.9%)	

Association (assessed by Chi square test) between targeted audience and healthcare professional or urological association involvement in the app development.

Moreover, there was statistically significant differences between apps’ type and HCP (p<0.001) and urological association (p<0.001) involvement in apps' design. The lack of HCP involvement in app development was highest in Patient Information apps (64.3%) and Electronic Medical Record/Diaries (40%). No Electronic Medical Record/Diaries nor Patient Information apps were developed with documented involvement by any scientific Urology association (Table 4).

**Table 4:** Association between type of application and expert involvement.

	Healthcare professional involvement in app’ development			Scientific Urology association involvement in app’ development		
Apps’ type	No (0)	Yes (1)	p-value	No (0)	Yes (1)	p-value
1-Reference, Guidelines, Quiz/Exam	4 (7.3%)	51 (92.7%)	0.000	44 (80%)	11 (20%)	0.000
2-Conferences, Urological Societies/Associations, Journals and Institutions	1 (2.3%)	43 (97.7%)		6 (13.6%)	38 (86.4%)	
3-Calculator	4 (30.8%)	9 (69.2%)		10 (76.9%)	3 (23.1%)	
4-Electronic Medical Record/Diaries	4 (40%)	6 (60%)		10 (100.0%)	0 (0.0%)	
5-Patient Information	18 (64.3%)	10 (35.7%)		28 (100.0%)	0 (0.0%)	

Association (assessed by Chi square test) between the type of application and the involvement of a healthcare professional or a scientific Urology association in the development of the app.

There were no statistically significant differences between target audience and cost (free or paid, p = 0.13) nor developer type (individual or organization, p = 0.08).

## Discussion

To our knowledge, this is the first study that completely identifies healthcare professional involvement in apps specifically designed for Urology and hence can serve as a trigger for urological societies to further explore the opportunities and overcome potential pitfalls of mHealth in Urology.

The mHealth market is mostly self-regulated, but there is a need for an independent assessment of available apps, to prevent both HCP and the general public from apps of using questionable reliability. The current study shows that there is clearly a deficit of expert input in Urology apps, as more than one fifth of all available apps did not have any involvement of HCP and only a third had the involvement of a scientific Urology association. These results are slightly worse than those from other areas, but this seems to be an issue across multiple medical fields [20–22].

Google Play Store has more Urology apps than Apple App Store. One possible explanation for this difference is the contrasting app approval process on both platforms: Android apps are automatically approved. However, iOS apps need to respect Apple's Review Guidelines and are only published in the App Store after technical approval by Apple staff [4]. For example, apps that share personal data without user consent are rejected [4].

The present study shows that the most common type of apps was "References, Guidelines, and Quiz/Exam" and most apps were available in the "Medical" category of the store, which is consistent with other reviews [13, 20].

Even though most apps were targeted at professionals, one fifth off all apps in our review are designed for patient information, which stresses the importance of safety even further, knowing that these users often lack scientific judgment.

Moreover, the involvement of commercial companies in this type of media has been questioned before, with worries about their funding and purpose [23]. A particular concern is the potential bias in product promotion, which can ultimately create conflicts of interest between the developer, healthcare professionals and the end user.

Development of health apps should always involve healthcare experts. However, in our review, medical calculators had an unexpectedly low proportion of HCP participation in their design. This lack of involvement is also evident in patient-targeted apps, particularly in Electronic Medical Records and Patient Information apps.

Even when there was reference to a HCP or an urological society involvement in the app design, it was not possible to systematically assess their level of responsibility, either as an external advisor, major stakeholder or sole author. Moreover, we could not find any available tools or evidence on how to quantify this information in a reproducible method.

This issue has raised attention of some public entities, namely the National Health Service in England, which curates an online Health Apps Library, and also private companies (e.g. Happtique MobileHealth Source), which are developing certification processes for mobile apps, with the aim of regulating the mHealth market [24].

With the growing number of available apps, the challenge is finding safe and well-designed apps. HCP and scientific Urology association involvement can act as a quality check, which is of paramount importance, not only for healthcare-targeted apps, but even more so in apps designed for the general public.

In the same way that doctors involvement in social media (SoMe) has been the focus of attention and recommendation by urological societies, medical app development should become subject to the same regulation, and Urology must not be left out [25–29].

mHealth has the potential to be an important tool in the future of Urology. However, it is critical that scientific accuracy, patient privacy and user safety are assured. Even though some of the issues may not be within the competence of scientific Urology associations, they can still take an active role in this subject.

mHealth app development should be seen by urologists as an opportunity to provide greater care to our patients and better software and knowledge to our peers. Even though this paradigm might require learning some new tools and skills, engaging in app development can be a fulfilling opportunity for a alternative medical interaction.

The present study certifies the lack of healthcare professional involvement in Urology apps. Considering that apps included in this review represent less than 0.2% of available medical apps and that there are more breast cancer (total = 178, Android = 118, iOS = 59) apps than Urology apps in total, mHealth is an untapped potential in our field and further investigation is mandatory to clarify the role that apps may play in Urology [30].

This study has some limitations. We could not perform analyses on the mobile stores' rating and review data because, unlike Google Play Store, the Apple App Store does not show the rating of all apps. Another limitation is that only the Android and iOS apps were reviewed, even though there are other mobile app stores, namely Microsoft and Samsung. However, Apple's App Store and Google's Play Store are by far the most popular platforms. We only searched for apps that included "Urology" in their metadata. Therefore, some Urology apps, which did not include it in the description of the app, were not included. Even though all graders had to agree on the app's type classification, it remains subjective, which is a potential limitation. However, there is no standardized classification scheme available. Healthcare professional and urological society involvement was structured as a binary variable (i.e. yes/no), but was not quantified.

## Conclusion

Apps represent a new opportunity to enhance care in Urology. Possible uses range from augmented reality apps that can be helpful in a clinical or surgical setting, to electronic diaries that aid in treatment monitoring and even health promoting apps. Even though there are, at the moment, 150 Urology apps, covering a wide range of subjects and directed at a diverse audience, there is room for improvement.

Until clear regulation is enforced, either by government health authorities or independent organizations, the urological community should adopt an active role as soon as possible, in a manner similar to what was done regarding SoMe [25–28]. Even though it is impossible to verify all available apps, maintaining a peer-reviewed register of certified Urology apps or issuing an official scientific seal of approval, could influence overall app design and, consequently, improve urological mHealth solutions.

## Supporting Information

S1 Table List of all included Urology apps. A complete assessment of all urology apps, including its description and information about its creators, is available as supporting information, and can be accessed at http://dx.doi.org/10.6084/m9.figshare.1363120
(DOC)
