# Supplementary material for: mHealth in Urology: A Review of Experts’ Involvement in App Development
Source: PLoS One. 2015 May 18;10(5):e0125547. doi: 10.1371/journal.pone.0125547 (PMC4436179; doi:10.1371/journal.pone.0125547)
Supplement: S1 Table — A complete assessment of all urology apps, including its description and information about its creators, is available as supporting information, and can be accessed at http://dx.doi.org/10.6084/m9.figshare.1363120 (DOC) [file pone.0125547.s001.doc]

### Supporting Information

### Table 1. List of all included Urology apps

| ***App Name*** | ***Mobile Platform*** | ***Scientific Urology Society involvement*** | ***Healthcare Professional involvement*** | ***Authors and Qualifications*** | ***App Type*** | ***App Description*** | ***Target Audience*** |
| --- | --- | --- | --- | --- | --- | --- | --- |
| @Hand: Urology | iOS | No | Yes | Associate Editor: Thomas D. DuBose, Jr., MD, MACP ; Tinsley R. Harrison Professor and Chair; Department of Internal Medicine;  Wake Forest University School of Medicine; Winston-Salem, North Carolina; Editor: Bruce F. Scharschmidt, M.D.; Senior Vice President and Chief Medical Officer; Hyperion Therapeutics, South San Francisco, CA | GRE | Provides medical reference information. | HCP |
| 28 Congreso de urologia 2014 | Android | Yes | Yes | Chilean Society of Urology | CJIUC | Official app of the Dominican National Society XXVIII Urology 2014 Congress | HCP |
| 3 Prostate Diseases (Tanzania) | Android | No | No | Albert Stone (Dev) | PI | Provides information about prostate diseases. | GP |
| Abnormal Urine Guide | iOS | No | No | Alekesey Kostin (Dev) | PI | Provides information about urine alterations. | GP |
| Advanced Urology | Android and iOS | No | No | Simpaddico (Dev) | GRE | Flashcards about Urology. | HCP |
| AUA 2011 Courses | Android | Yes | Yes | American Urological Association | CJIUC | AUA 2011 official app. | HCP |
| AUA 2012 Annual Meeting | Android | Yes | Yes | American Urological Association | CJIUC | AUA 2012 official app. | HCP |
| AUA 2013 Annual Meeting | Android and iOS | Yes | Yes | American Urological Association | CJIUC | AUA 2013 official app. | HCP |
| AUA 2014 Annual Meeting | Android and iOS | Yes | Yes | American Urological Association | CJIUC | AUA 2014 official app. | HCP |
| AUA Annual Meeting | Android | Yes | Yes | American Urological Association | CJIUC | Provides information about the AUA Meeting. | HCP |
| AUA Core Curriculum Mobile | Android and iOS | Yes | Yes | American Urological Association | GRE | Provides medical reference information. | HCP |
| AUA EBJC - Evidence-Based JC | Android | Yes | Yes | American Urological Association | CJIUC | Provides medical reference information. | HCP |
| AUA Guidelines at a Glance | Android | Yes | Yes | American Urological Association | GRE | AUA Guidelines offical app. | HCP |
| AUA Medical Student Curriculum | Android and iOS | Yes | Yes | American Urological Association | GRE | Helps users learn the core principles and practices in urology. | HCP |
| AUA Member Search | Android and iOS | Yes | Yes | American Urological Association | CJIUC | Directory of AUA members. | HCP |
| AUA Men's Health Checklist | Android | Yes | Yes | American Urological Association | GRE | Provides medical reference information. | HCP |
| AURO.it Nazionale 2013 | iOS | Yes | Yes | Italian Association of Urology | CJIUC | AURO.it Nazionale 2013 official app | HCP |
| Bedwetting Info | Android | No | No | BAWidgets.com (Dev) | PI | Provides recommendations about bedwetting. | GP |
| Bedwetting solutions | Android | No | No | Mittie (Dev) | PI | Provides recommendations about bedwetting. | GP |
| Bedwetting Trainer | Android | No | Yes | Dr. Kazuhiro Tajima and TKT Brain Solutions | PHR | Provides recommendations about nocturia. | GP |
| BJUI Journal | iOS | Yes | Yes | BJU International | CJIUC | BJUI Journal official app. | HCP |
| Bladder Cancer Prognosis Calc | iOS | Yes | Yes | European Organisation for Research and Treatment of Cancer | Calculator | Predicts recurrence and progression of Ta and T1 bladder cancer. | HCP |
| Bladder Pal | Android | No | Yes | Dr. R Yap, Urologist | PHR | Digital voiding diary. | GP |
| Braz J Urol | Android and iOS | Yes | Yes | Brazilian Society of Urology | CJIUC | Brazilian Journal of Urology official app. | HCP |
| Briganti Nomogram | Android | No | No | Anders Bisbjerg (Dev) | Calculator | Risk calculator based on the "Briganti nomogram EPLD 2012". | HCP |
| BSC Urology Events | Android and iOS | No | No | CrowdCompass (Dev) | CJIUC | List of events about Urology and Women's Health | HCP |
| CalcuLithiasis | iOS | Yes | Yes | French Association of Urology | Calculator | Urolithiasis risk calculator. | HCP |
| CAU2014 | Android | Yes | Yes | American Confederation of Urology | CJIUC | CAU2014 | HCP |
| CROJ | Android | No | Yes | Dr. F Dal Moro, Urologist | CJIUC | CASE REPORT ONLINE JOURNAL official app. | HCP |
| CRPC Nomogram App | Android | No | Yes | Andrew J.Armstrong MD ScM, Elizabeth S. Garrett-Mayer PhD, Yi-Chun Ou Yang PhD, Ronald de Wit MD, Ian F.Tannock MD,and Mario Eisenberger MD | Calculator | Calculates estimated survival rates among men with metastatic castration resistant prostate cancer. | HCP |
| CURE-UAB | Android and iOS | No | Yes | Accreditation Council for Continuing Medical Education | CJIUC | Congress of Urological Research and Education on Aging Underactive Bladder official app. | HCP |
| Current Opinion in Urology | iOS | No | Yes | Editorial Board | CJIUC | Current Opinion in Urology official app. | HCP |
| Daily-P | Android and iOS | No | Yes | Dr. N Pereira-Azevedo, Urologist | PHR | Digital voiding diary. | GP |
| Daily-P Pro | Android and iOS | No | Yes | Dr. N. Pereira-Azevedo, Urologist | PHR | Allows HCP to connect with patients and manage voiding diaries. | HCP |
| Dealing with Prostate Cancer | Android | No | No | Vertex Mind (Dev) | PI | Provides information about Prostate Cancer. | GP |
| Dealing with Prostate Cancer Free | Android | No | No | Vertex Mind (Dev) | PI | Provides information about Prostate Cancer. | GP |
| DGU 2012 | Android and iOS | Yes | Yes | [German Society of Urology](http://www.emiratesurology.com/EUS/deutsch-gesellschaft-fur-urologie-dgu/) | CJIUC | DGU 2012 official app. | HCP |
| DGU 2014 | iOS | Yes | Yes | [German Society of Urology](http://www.emiratesurology.com/EUS/deutsch-gesellschaft-fur-urologie-dgu/) | CJIUC | DGU 2014 official app. | HCP |
| DGU 2014 - Kongress App | Android | Yes | Yes | [German Society of Urology](http://www.emiratesurology.com/EUS/deutsch-gesellschaft-fur-urologie-dgu/) | CJIUC | DGU 2014 official app. | HCP |
| drawMD Female Pelvic Surgery | iOS | No | Yes | Dr. J Tan-Kim, M.D. | GRE | Allows patient education by drawing on medical illustrations. | HCP |
| drawMD Urology - Patient Education by Drawing on Medical | iOS | No | Yes | Dr. A Kutiko, Urologist | GRE | Allows patient education by drawing on medical illustrations. | HCP |
| DutasT | Android and iOS | No | Yes | HCP of Magna Health Solutions | GRE | Dutas Urology Update | HCP |
| e-URO Tools | Android and iOS | Yes | Yes | European Association of Urology | GRE | EAU-Italian-British-French Guidelines and Urological Tools for the daily practice in Urology. | HCP |
| EAU 2012 | Android and iOS | Yes | Yes | European Association of Urology | CJIUC | EAU 2012 official app. | HCP |
| EAU Milan 2013 | Android and iOS | Yes | Yes | European Association of Urology | CJIUC | EAU 2013 official app. | HCP |
| EAU Pocket Guidelines | Android and iOS | Yes | Yes | European Association of Urology | GRE | EAU Guidelines official app. | HCP |
| EAU Stockholm 2014 | Android and iOS | Yes | Yes | European Association of Urology | CJIUC | EAU 2014 official app. | HCP |
| EAU Vienna 2011 | Android and iOS | Yes | Yes | European Association of Urology | CJIUC | EAU 2011 official app. | HCP |
| EAUN Milan 2013 | Android and iOS | Yes | Yes | European Association of Urology | CJIUC | EAUN 2013 official app. | HCP |
| EAUN Stockholm 2014 | Android and iOS | Yes | Yes | European Association of Urology | CJIUC | EAUN 2014 official app. | HCP |
| ESPU 2012 | Android and iOS | Yes | Yes | European Society for Paediatric Urology | CJIUC | ESPU 2012 official app. | HCP |
| ESPU 2013 | Android and iOS | Yes | Yes | European Society for Paediatric Urology | CJIUC | ESPU 2013 official app. | HCP |
| European Urology app | Android and iOS | Yes | Yes | European Association of Urology | CJIUC | European Urology official app. | HCP |
| EurUro SiM | iOS | Yes | Yes | European Association of Urology | GRE | Surgical videos and animations. | HCP |
| Female Pelvic Medicine & Reconstructive Surgery | iOS | Yes | Yes | American Urological Association | CJIUC | Female Pelvic Medicine & Reconstructive Surgery official app. | HCP |
| Foundation Urology | Android | No | Yes | Dr. J Durrant and Dr. F Lee, Urologists | GRE | Provides reference medical information. | HCP |
| GU Path I | iOS | No | Yes | Dr. D Ramnani, Pathologist | GRE | Provides reference medical information. | HCP |
| GU Path Lite | iOS | No | Yes | Dr. D Ramnani, Pathologist | GRE | Provides reference medical information. | HCP |
| HapPee Time | Android | No | No | Helpful Animals (Dev) | PHR | Digital voiding diary. | GP |
| iCU Evora 2011 | iOS | Yes | Yes | Dr. N Pereira-Azevedo, Dr. E Carrasquinho and Dr. E Cardoso de Oliveira, Urologists | CJIUC | iCU Evora 2011 official app- | HCP |
| iDry | iOS | No | Yes | Dr. J Nelson, Urologist | PHR | Digital voiding diary. | GP |
| Int'l Urogynecology Journal | Android | Yes | Yes | International Urogynecological Association | CJIUC | The International Urogynecology Journal official app. | HCP |
| iP Voiding Diary | iOS | No | Yes | Erich Taubert, Urologist | PHR | Digital voiding diary. | GP |
| iReflux Risk Calculator | Android and iOS | No | No | PaperAct Solutions (Dev) | Calculator | Risk calculator for urinary tract infections in children with primary vesicoureteral reflux. | GP |
| itsaMANTHING - Prostate Cancer | Android | No | No | PROSTaid (Dev) | PI | Provides information about Prostate Cancer. | GP |
| iURO Andrology | Android and iOS | No | Yes | Dr. E Garcia, Dr. J Salamanca, Dr. I Moncada, Dr. J Rioja and Dr. M Servet, Urologists | GRE | Provides medical reference information about Andrology. | HCP |
| iURO Andrology PRO | Android and iOS | No | Yes | Dr. E Garcia, Dr. J Salamanca, Dr. I Moncada, Dr. J Rioja and Dr. M Servet, Urologists | GRE | Provides medical reference information about Andrology. | HCP |
| iURO General Practicioner | iOS | No | Yes | Dr. O Traxer, Dr. R Parra, Dr. E Garcia, Dr. J de la Rosette, Dr. C Rioja | GRE | Provides medical reference information. | HCP |
| iURO Kidney | Android and iOS | No | Yes | Dr. O Traxer and Dr. C Rioja, Urologists | GRE | Provides medical reference information about kidney diseases. | HCP |
| iURO Oncology | Android and iOS | No | Yes | Dr. J Rioja, Dr. C Rioja, Dr. K Touijer, Dr. R Parra, Dr. P Scardino, Dr. P Laguna, Urologists | GRE | Provides medical reference information about urological oncology. | HCP |
| iURO Oncology Pro | Android and iOS | No | Yes | Dr. J Rioja, Dr. C Rioja, Dr. K Touijer, D. P Scardino, Dr. P Laguna, Urologists | GRE | Provides medical reference information about urological oncology. | HCP |
| iURO Pelvic Floor | Android and iOS | No | Yes | Dr. C Rioja, Urologist | GRE | Provides medical reference information about pelvic floor pathologies. | HCP |
| iURO Pelvic Floor Pro | Android and iOS | No | Yes | Dr. C Rioja, Urologist | GRE | Provides medical reference information about pelvic floor pathologies. | HCP |
| iURO Prostate Pro | Android and iOS | No | Yes | Dr. J de la Rosette, Dr. C Rioja, Dr. J Rioja, Urologists | GRE | Provides medical reference information about prostate diseases. | HCP |
| Kidney and Bladder Problems | Android | No | No | KoolAppz (Dev) | PI | Provides information about kidney and bladder problems. | GP |
| Kidney Cancer | Android | No | No | VPV (Dev) | PI | Raises awareness for the Kidney Cancer Association. | GP |
| Kidney Disease Assistant | iOS | No | No | Deerane He (Dev) | PHR | Provides information about kidney disease. | GP |
| Kidney Diseases | iOS | No | No | Dilip Bhagora (Dev) | GRE | Provides information about kidney disease. | HCP |
| Kidney Urology - Simulations and behaviours of Kidney diseases | iOS | No | Yes | Dr. O Traxer, Dr. C Rioja, Dr. J Rioja, Urologists | GRE | Provides medical reference information about kidney diseases. | HCP |
| kidneystoneMD | iOS | No | Yes | “This app was developed by a practicing urologist.” | PI | Provides medical reference information about kidney stones. | GP |
| Learning Urology Quiz | Android and iOS | No | Yes | HCP of [Learning Nurse Resources Network (Dev)](http://www.itrdonline.com/www.learningnurse.com/index.php/assessment/apps) | GRE | Flashcards about urology. | HCP |
| Male impotence risk evaluation | Android | No | No | Andrea Ciarrocchi (Dev) | Calculator | Risk calculator for erectile dysfunction. | GP |
| male_Japanese | iOS | No | Yes | Dr. M Oishi, Urologist | PI | Provides questions about sexually transmitted diseases. | GP |
| Men's App | iOS | No | Yes | “It has been created by expert urologists.” | PI | Provides information about male urological diseases. | GP |
| Men's Guide To Prostate Health | Android | No | No | KoolAppz (Dev) | PI | Provides information about prostate diseases. | GP |
| Miniatlas Erectile Dysfunction | iOS | No | Yes | [Amit Patel,](http://www.imedicalapps.com/author/amit/) Urologist | GRE | Provides information and high quality pictures about erectile dysfunction. | HCP |
| My BladderDiary | Android | No | No | ACORN Technologies (Dev) | PHR | Digital voiding diary. | GP |
| NMIBC Toolbox | Android | No | Yes | Medac (Parmaceutical company) (Dev) | Calculator | Provides information about non-muscle invasive Bladder Cancer, based on the EAU guidelines. | HCP |
| Oxford Handbook Urology 2nd Ed | Android and iOS | No | Yes | Dr. J Reynard, Dr. S Brewster, Dr. S Biers, Urologists | GRE | Provides medical reference information about general urology. | HCP |
| Partin/Han Tables | iOS | No | Yes | Dr. A Partin, Dr. P Walsh, Dr. M Han, Urologists | Calculator | Predicts the probability of detectable PSA following radical surgery. | HCP |
| PI-RADS Prostate MRI | Android | No | Yes | Dr. L Lassalle, Urologist | Calculator | Allows PI-RADS scoring of lesions in prostate multiparametric MRI according to the european society of uro-radiology (ESUR) 2012 guidelines. | HCP |
| Prac. Urology for Primary Care | Android and iOS | No | Yes | Dr. E Rico, Urologist | GRE | Provides urological reference information. | HCP |
| Practical Urology | Android and iOS | No | Yes | Dr. E Rico, Urologist | GRE | Provides urological oncology reference information. | HCP |
| Practical Urology for Gynecologists (iPhone version) | iOS | No | Yes | Dr. E Rico, Urologist | GRE | Provides urological oncology reference information. | HCP |
| Prevent Prostate Cancer | Android | No | No | Graham Player, Ph.D. (Health Sciences) | PI | Provides nutritional recommendations to prevent prostate cancer. | GP |
| Primary Care Guidelines for Urology | Android and iOS | Yes | Yes | American Urological Association | GRE | AUA Primary Care Guidelines for Urology official app. | HCP |
| Prostate Cancer | Android | No | Yes | HCP of Magna Health Solutions | PI | Provides general information about prostate cancer. | GP |
| Prostate Cancer Calculator | Android | No | No | Borinfer (Dev) | Calculator | Calculates the risk of prostate cancer. | GP |
| Prostate Cancer Calculator (Seoul National University) | Android | No | Yes | Dr. C Jeong, Dr. S Lee, Dr. J Jung, Dr. B Lee, Dr. S Jeong, Dr. S Hong, Dr. S Byun, Dr. S Lee, Urologists | Calculator | Prostate cancer risk calculator. | HCP |
| Prostate Cancer v2 | Android | No | Yes | Dr. S Tabatabaei, Urologist | PI | Provides nutritional recommendations to prevent prostate cancer. | GP |
| Prostate Health | Android and iOS | No | Yes | Dr. E Rico, Urologist | PI | Provides general information about prostate diseases. | GP |
| Prostate In Focus | Android | No | Yes | HCP of Magna Health Solutions | GRE | Provides reference information about prostate cancer. | HCP |
| PROSTATE INTERNATIONAL | Android | Yes | Yes | Asia Pacific Prostate Society | CJIUC | Journal of Asia Pacific Prostate Society official app. | HCP |
| Prostate Pal 2 | Android | No | Yes | Dr. R Yap, Urologist | PI | Keeps a record of the patient’s clinical evaluations and exams. | GP |
| ProstateMD | Android | No | Yes | Dr. J McHugh and Dr. McHugh, Urologists | PI | Provides general information about prostate cancer. | GP |
| Renal & Urology News | Android and iOS | No | Yes | [Dr. R Uzzo, Urologist](http://www.renalandurologynews.com/editorial-board/section/46/) | GRE | Provide access to clinical news, CME courses, medical conference highlights, and other resources. | HCP |
| Reviews in Urology | iOS | No | Yes | Dr. M Brawer, MD | GRE | Reviews the latest advances in the diagnosis and treatment of a wide range of urological conditions. | HCP |
| Rotterdam Prostate Cancer Risk Calculator | Android and iOS | Yes | Yes | Prostate Cancer Research Foundation (SWOP) and Dr. N Pereira-Azevedo | Calculator | Rotterdam Prostate Cancer Risk Calculator official app. | GP |
| РОУ (Russion Society of Urology) | Android | Yes | Yes | Russian Society of Urology | CJIUC | Russian Society of Urology official app. | HCP |
| Show Me OAB | iOS | No | No | Astellas (Dev) | PI | Interactive clinical tool designed to facilitate dialogue between HCP and their patients dealing with overactive bladder. | GP |
| SIU 2013 | Android and iOS | Yes | Yes | Société Internationale d'Urologie | CJIUC | SIU 2013 official app. | HCP |
| SMU 2014 | Android | Yes | Yes | Mexican Urology Association | CJIUC | SMU 2014 official app. | HCP |
| Testicle pain, testicle tumors | Android | No | No | ConstantaSoft (Dev) | PI | Provides information about testicle pain and cancer. | GP |
| Testicular Cancer | Android | No | No | Spence (Dev) | PI | Provides information about testicle cancer. | GP |
| Testicular Cancer Checker | iOS | No | No | PSJ Investments (Dev) | PI | Provides information about testicle cancer. | GP |
| The Journal of Urology®, Official Journal of AUA | iOS | Yes | Yes | American Urological Association | CJIUC | Official app of the Journal of the American Urological Association. | HCP |
| Three Diseases of the Prostate | Android | No | No | Albert Stone (Dev) | PI | Provides information about prostate diseases. | GP |
| TNM Urology | iOS | No | Yes | American Joint Committee on Cancer | Calculator | Official app of the 7th Edition of the AJCC Cancer Staging Manual. | HCP |
| Turkish Journal of Urology | iOS | Yes | Yes | Turkish Association of Urology | CJIUC | Official app of the Turkish Journal of Urology. | HCP |
| Understanding Prostate Cancer | Android | No | Yes | HCP of Springer Healthcare | GRE | Provides reference information about prostate cancer. | HCP |
| UrinaryAlmanac | iOS | No | Yes | HCP of iMedAppsTeam | GRE | Provides reference information about urology. | HCP |
| Uro Challenge | Android and iOS | Yes | Yes | American Urological Association | GRE | Provides reference information about urology. | HCP |
| UroBladderDiary | iOS | No | No | William Carithers (Dev) | PHR | Digital voiding diary. | GP |
| Urolithiasis Assist | Android | Yes | Yes | European Association of Urology | GRE | European Association of Urology Urolithiasis Guidelines official app. | HCP |
| Urologic Nurse CURN, 800 MCQs | Android | No | Yes | Physicians and nurses from Knowledge Testing, Inc. (Dev) | GRE | Multiple choice questions about Urology. | HCP |
| Urologic Oncology: Seminars and Original Investigation | iOS | Yes | Yes | Society of Urologic Oncology | CJIUC | Official app of the Journal of the Society of Urologic Oncology. | HCP |
| Urological Surgery | Android | No | Yes | Dr. J Reynard, Dr. M Sullivan, Dr. S Mark, Dr. K Turner, Dr. N Armenakas, UrologistsAndroid | CJIUC | Oxford Specialist Handbook of Urological Surgery official app. | HCP |
| Urological Ultrasound | Android | No | Yes | HCP of Ultrasoundpaedia | GRE | Description on how to perform urological ultrasound. | HCP |
| Urology | iOS | No | No | Armend Qushku (Dev) | GRE | Contains videos of surgical interventions and information about urological drugs. | HCP |
| Urology - Pediatric, 1000 MCQs | Android | No | Yes | HCP of Knowledge Testing | GRE | Multiple choice questions about urology. | HCP |
| Urology Board Review Manual | Android and iOS | No | Yes | HCP of Turner White | GRE | Gives you full-text access to the Hospital Physician Urology BRM. | HCP |
| Urology Case Reports | iOS | Yes | Yes | HCP of Elsevier Inc. | CJIUC | Official app of the Journal of Urology Case Reports. | HCP |
| Urology Flashcards | Android and iOS | No | Yes | Dr R Ramasamy and Dr. G Kalligal, Urologists | GRE | App with over 1500 flashcards about urology. | HCP |
| Urology for Gynecologists | Android | No | Yes | Dr. E Rico, Urologist | GRE | Provides reference information about urology. | HCP |
| Urology Glossary | Android and iOS | No | No | Publish This (Dev) | GRE | Provides general information about urology. | HCP |
| Urology Nation | Android and iOS | No | Yes | Dr. R Mordkin, MD, Dr G Bernstein, Dr. A Joel, Urologists | CJIUC | Urology Nation official app. | HCP |
| Urology NBI Atlas by Olympus | iOS | No | Yes | Olympus (Dev) | GRE | Allows clinicians to view different image sets in white light and NBI light to advance their understanding of endoscopic imaging using Narrow Band Imaging (NBI) technology. | HCP |
| Urology Patient Education by CoherentRx | iOS | No | Yes | HCP of Coherentrx | GRE | Pairing interactive 3D anatomy models with a HIPAA-compliant doctor-patient messaging tool. | GP |
| Urology Planet | iOS | No | No | Edizione Manent (Dev) | PI | Provides general information about urology. | GP |
| Urology Times | Android and iOS | No | Yes | HCP of Urology Times | CJIUC | Urology Times official app. | HCP |
| Urology, 1000 MCQs | Android | No | Yes | HCP of Knowledge Testing (Dev) | GRE | 1000 multiple choice questions about urology. | HCP |
| Urology, The Gold Journal | iOS | Yes | Yes | Société Internationale d'Urologie | CJIUC | Official app of Urology, the Gold Journal. | HCP |
| UrologyMatch | Android and iOS | No | Yes | HCP of Urology Match | CJIUC | UrologyMatch official app. | HCP |
| UroSketch 3D Explore | iOS | No | Yes | HCP of GenomeDx Biosciences | PI | Provides an interactive 3D model of the prostate, bladder and surrounding organs for use by patients. | GP |
| UroSketch 3D Professional | iOS | No | Yes | HCP of GenomeDx Biosciences | GRE | Provides a 3D model of the prostate, bladder and surrounding organs for use by physicians during consult with their patients. | HCP |
| USICON | Android | Yes | Yes | Urological Society of India | CJIUC | Annual Conference of Urological Society of India 2013 official app. | HCP |
| USICON 2014 | Android and iOS | Yes | Yes | Urological Society of India | CJIUC | Annual Conference of Urological Society of India 2014 official app. | HCP |
| UWPEN | iOS | No | No | Seattle AppLab (Dev) | PI | Allows men that suffer from Peyronie's disease to perform penile asymmetry measurements. | GP |
| Vasectomy Reversal | Android | No | Yes | Dr. M Glick, M.D. | PI | Provides general information about vasectomy. | sGP |
| WCE 2013 Annual Meeting | Android | Yes | Yes | Endourological Society | CJIUC | WCE 2013 official app. | HCP |

**Abbreviations used:**

**AUA** – American Urological association;

**CJIUC –** Conferences, Journals, Institution and Urological Society;

**EAU** – European Urological Association;

**GP –** General public;

**GRE –** Guidelines, references and exam;

**HCP –** Healthcare professionals;

**PHR –** Electronic Medical Record / Diary;

**PI –** Patient Information.
